# Supplementary material for: Venomix: a simple bioinformatic pipeline for identifying and characterizing toxin gene candidates from transcriptomic data
Source: PeerJ. 2018 Jul 31;6:e5361. doi: 10.7717/peerj.5361 (PMC6074769; doi:10.7717/peerj.5361)
Supplement: Supplemental Information 3 [file peerj-06-5361-s003.gz › FinalOutput_E-6/Hyaluronidase_CdtHya1_1/finaltree.pdf]

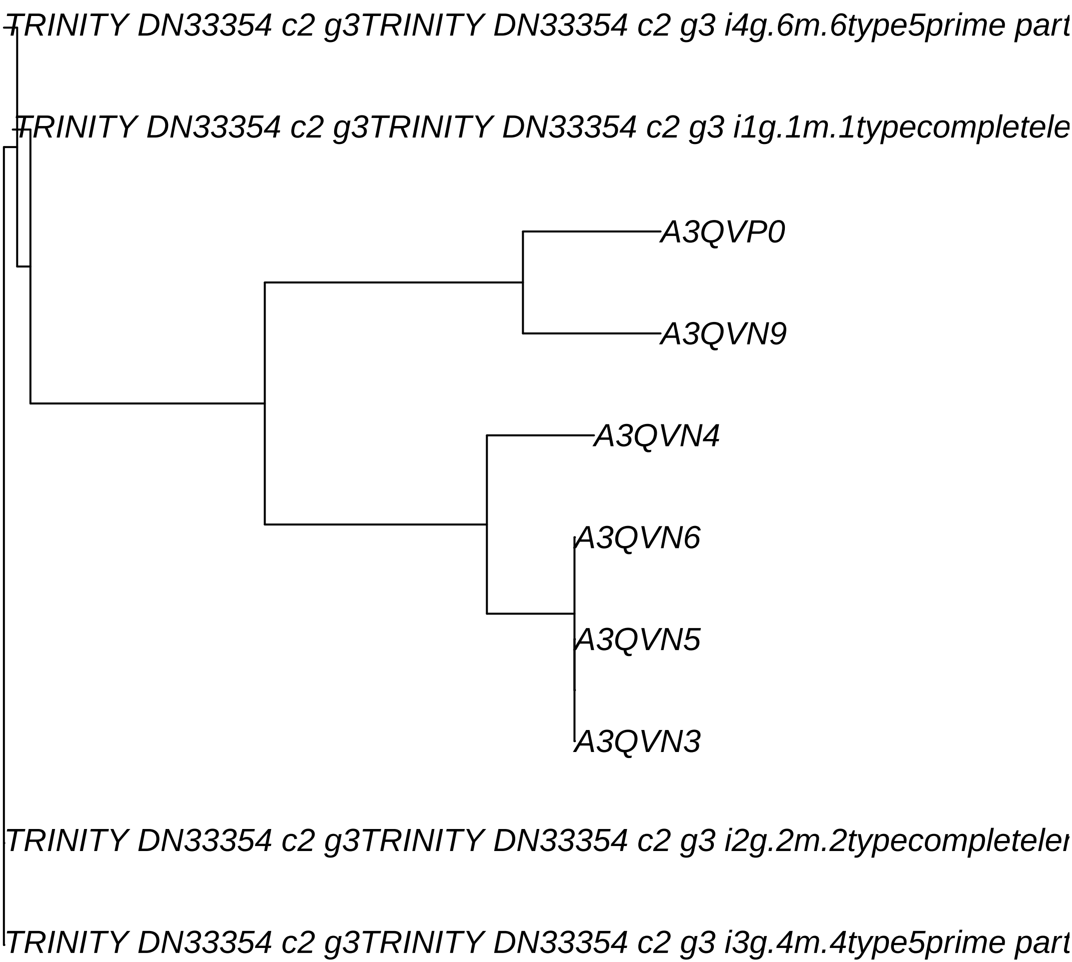
TRINITY DN33354 c2 g3TRINITY DN33354 c2 g3 i4g.6m.6type5prime part
TRINITY DN33354 c2 g3TRINITY DN33354 c2 g3 i1g.1m.1typecompletele
A3QVP0
A3QVN9
A3QVN4
A3QVN6
A3QVN5
A3QVN3
TRINITY DN33354 c2 g3TRINITY DN33354 c2 g3 i2g.2m.2typecompletele
TRINITY DN33354 c2 g3TRINITY DN33354 c2 g3 i3g.4m.4type5prime part
